# A comparison of commercial and custom-made electronic tracking systems to measure patient flow through an ambulatory clinic

**DOI:** 10.1186/s12942-015-0023-7

**Published:** 2015-10-29

**Authors:** Sharif Vakili, Ravi Pandit, Eric L. Singman, Jeffrey Appelbaum, Michael V. Boland

**Affiliations:** Johns Hopkins University School of Medicine, Baltimore, MD USA; Wilmer Eye Institute, Johns Hopkins University School of Medicine, Baltimore, MD USA; Johns Hopkins Bloomberg School of Public Health, Baltimore, MD USA; Operations Integration, Johns Hopkins Health System, Baltimore, MD USA; Division of Health Sciences Informatics, Johns Hopkins University School of Medicine, Baltimore, MD USA

**Keywords:** Radio-frequency identification (RFID), Real-time locating systems (RTLS), Infrared (IR) tracking, Patient flow, Patient tracking, Healthcare management, Clinic management, Raspberry Pi, Centrak

## Abstract

**Background:**

Understanding how patients move through outpatient clinics is important for optimizing clinic processes. This study compares the costs, benefits, and challenges of two clinically important methods for measuring patient flow: (1) a commercial system using infrared (IR) technology that passively tracks patient movements and (2) a custom-built, low cost, networked radio frequency identification (RFID) system that requires active swiping by patients at proximity card readers.

**Methods:**

Readers for both the IR and RFID systems were installed in the General Eye Service of the Wilmer Eye Institute. Participants were given both IR and RFID tags to measure the time they spent in various clinic stations. Simultaneously, investigators recorded the times at which patients moved between rooms. These measurements were considered the standard against which the other methods were compared.

**Results:**

One hundred twelve patients generated a total of 252 events over the course of 6 days. The proportion of events successfully recorded by the RFID system (83.7 %) was significantly greater than that obtained with the IR system (75.4 %, p < 0.001). The cause of the missing events using the IR method was found to be a signal interruption between the patient tags and the check-in desk receiver. Excluding those data, the IR system successfully recorded 94.4 % of events (p = 0.002; OR = 3.83 compared to the RFID system). There was no statistical difference between the IR, RFID, and manual time measurements (p > 0.05 for all comparisons).

**Conclusions:**

Both RFID and IR methods are effective at providing patient flow information. The custom-made RFID system was as accurate as IR and was installed at about 10 % the cost. Given its significantly lower costs, the RFID option may be an appealing option for smaller clinics with more limited budgets.

## Background

Optimizing patient flow through ambulatory clinics is important for reducing wait times, minimizing health care costs, improving patient satisfaction and providing high quality health care [1]. In 2014, the average office wait time to see a physician was 20 min [2], with wait times that could be much longer, especially in urban areas. Additionally, surveys reveal that patient satisfaction decreases markedly with increased clinic wait times, particularly when waits exceed 20 min [3]. There is therefore a great need for interventions to reduce clinic wait times and make clinic operations more efficient.

Successful practice management necessitates assessments of patient flow so that the impact of interventions can be determined. As standard management theory reveals [4], it is not possible to effectively manage that which cannot be measured. Real-time locating systems (RTLS) allow for such assessments. RTLS include a variety of technologies for asset tracking, including Bluetooth, iBeacon, Wi-Fi, camera vision, ultrasound, radio frequency identification (RFID), infrared (IR), global positioning systems (GPS), and cellular signals [5]. Each of these technologies has its own strengths and methods of data acquisition [5, 6]. For example, some technologies, such as Wi-Fi and cellular triangulation, can capture signals from large areas and across walls but typically lack the location precision needed for many tracking situations [7]. Bluetooth technologies like iBeacon and satellite triangulation systems like GPS can provide more accurate localization but the former requires smartphone or Bluetooth-compatible device integration [8] and the latter has limited functionality indoors [9].

For patient flow measurements in clinic settings, the majority of RTLS methods incorporate RFID or IR technologies because these methods use designated transmitters and receivers for purportedly precise indoor room-level location information and involve setups suitable for clinics [10]. However, objective publications validating these technologies have been limited. Many published studies have either been conducted by industry or have been industry-sponsored [11–14].

Among non-industry sponsored research, we found only two studies evaluating commercial RTLS in real-world healthcare settings using human measurements as a reference [15, 16]. Both of these studies assessed IR tracking technology, and their results have been mixed. One looked at operating room (OR) patient tracking and reported 69 % more accurate documentation with IR methods than manual standards but also 15 % missing data (n = 93) [15]. Despite missing data, the investigators found their system to ultimately be useful. A separate study using researchers to simulate patients’ clinic movements had less favorable results [16]. Their IR system had an average location inaccuracy of 2 m with particularly unreliable location reporting in hallways (n = 84 h of data). Investigators reported that they had to recalibrate their system four times in their 3 months of testing to improve accuracy and ultimately found their current level of technology inadequate for precise clinic flow.

Other non-industry studies evaluating RTLS have been more qualitative. A 3-year study examining 23 US hospitals found that most RTLS systems had substandard functionality and serious obstacles to effective deployment [17]. Often, indoor location systems require manual tweaking to get satisfactory levels of accuracy [18], and signals from other equipment can cause interference [5, 6]. Costs have also been reported to be potentially significant barriers to ongoing use [19, 20]. For example, lost patient tags and installation of the infrastructure can cost thousands of dollars [1, 5, 19]. Nevertheless, significant cost savings have been suggested with proper use of the technology [5]. One group using surveys and interviews calculated that appropriate use of RTLS could save $750,000 at their hospital [21]. However, rigorous cost-benefit analysis in the literature is still lacking.

Given these potential limitations, it may be of use to investigate the feasibility and potential cost advantages of custom-built RFID systems. Nowhere in the literature have custom-built systems been investigated nor have methods been shared to open the use of such systems. A better understanding of these tracking technologies is important for physicians and administrators looking to invest in RTLS technologies for practice management. Therefore, to address this need, we conducted a study to validate an industry standard commercial IR system and a custom-built RFID system. The custom-built RFID system presented here is investigated as a potential alternative to industry standards.

## Methods

**Fig. 1:**
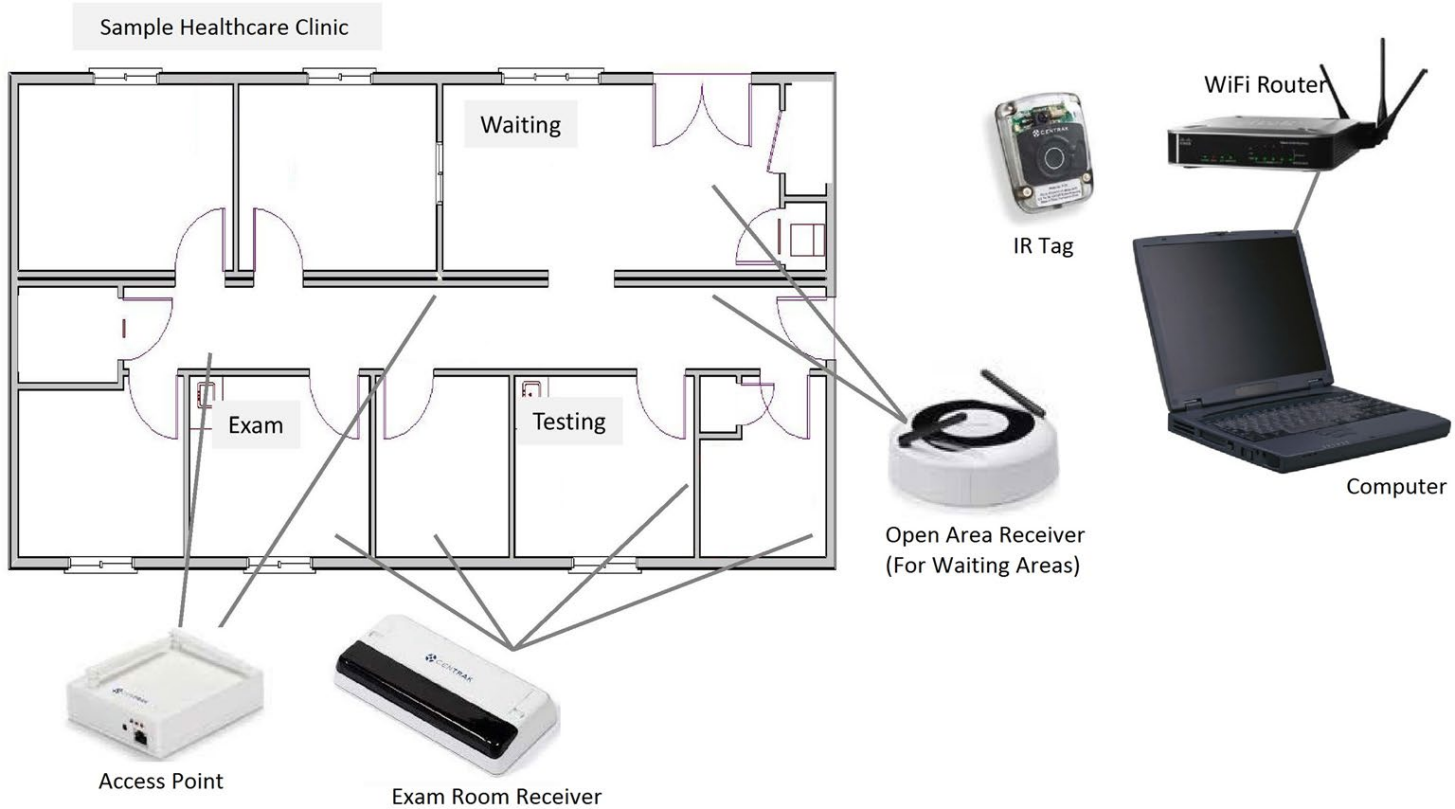
Infrared (IR) tracking system overview: (1) the patient is given a battery-powered IR tag. (2) The tag is activated by an infrared signal from exam room or open area receivers. (3) The tag emits a 900 MHz radiofrequency signal with tag number to access points. (4) Access points send the IR tag number and receiver location data to a computer via Wi-Fi routers. (5) A computer runs data collection software

The IR technology used was produced by CenTrak (CenTrak, Newtown, PA, USA). Receivers were installed in the ceilings of examination rooms, waiting rooms and the front desk, and access points installed in the ceilings accompanied a set of about 5 receivers (Fig. 1). Unique battery-powered tags received IR signals from transmitters and then transmitted 900 mHz radiofrequency (RF) signals to access points, which registered the tags’ locations with millisecond precision. The access points then transferred tag location information to a computer through a Wi-Fi network deployed solely for the IR system. The computer ran proprietary data collections software from CenTrak.

**Fig. 2:**
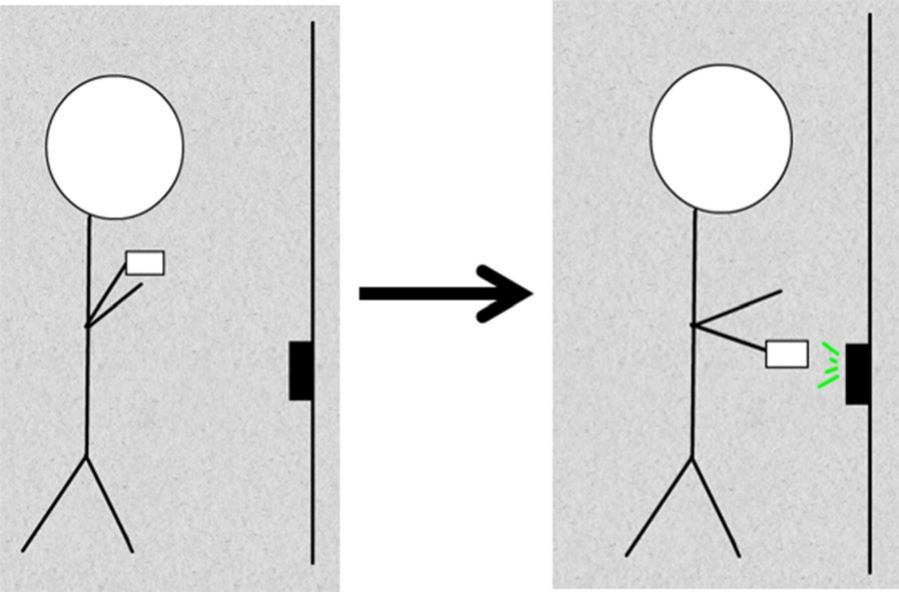
Radio frequency identification (RFID) system overview: (1) the patient is given an RFID tag. (2) The tag is swiped within a six-inch proximity of the radiofrequency card reader. (3) The reader LED light flashes confirming registration of the tag ID and swipe time

**Fig. 3:**
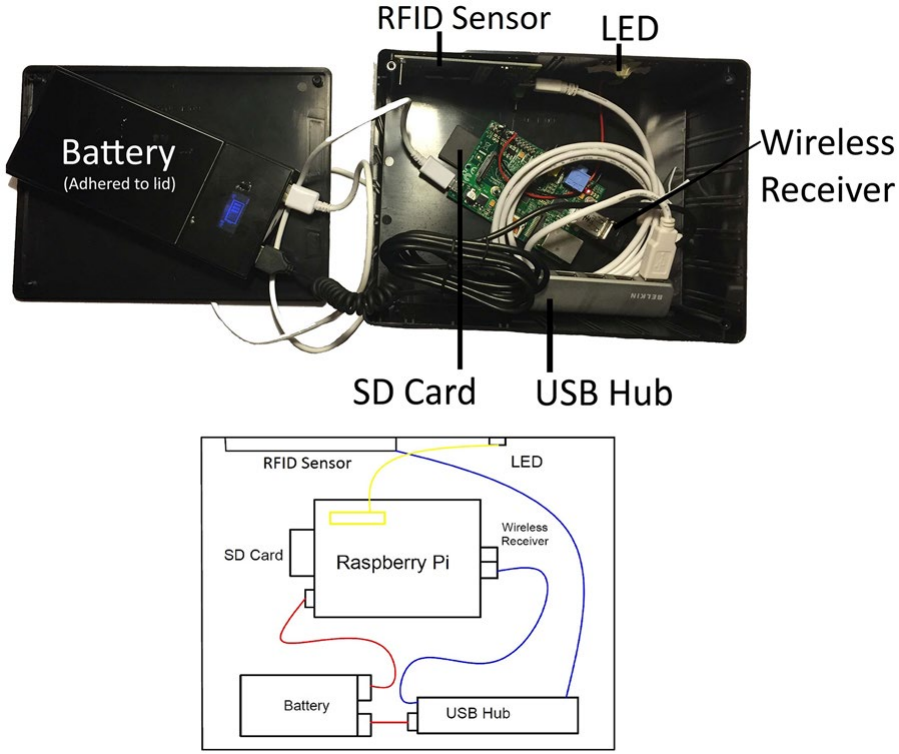
Schematic of custom-built RFID system: The schematic of the RFID receiver is shown. The project enclosure box encasing the system is 8 × 6 × 3″. It was enclosed using included screws and adhered beside the clinic door using removable industrial fastening strips

The RFID technology used in the study was custom-built by the investigators. The RFID reader was assembled using a Raspberry Pi minicomputer (Raspberry Pi, Caldecote, UK) linked to an RFID sensor (Phidgets, Calgary, Alberta) by custom programming. The device was built to be completely wireless and battery-powered so that it could be operated in a variety of clinic settings. The existing enterprise wireless network was used to access time servers for accurate time synchronization. The same network was also used to transmit data from the RFID readers to a central database for subsequent analysis. The readers emitted a radiofrequency signal detecting unique proximity cards as they were swiped. Timestamps registered to the second, card ID and sensor ID were recorded with each card swipe. Cards could be swiped at a distance of up to six inches away and installed LED lights on the reader provided feedback to the participants regarding successful card registration (Fig. 2). A schematic of the system has been provided for public use (Fig. 3).

**Fig. 4:**
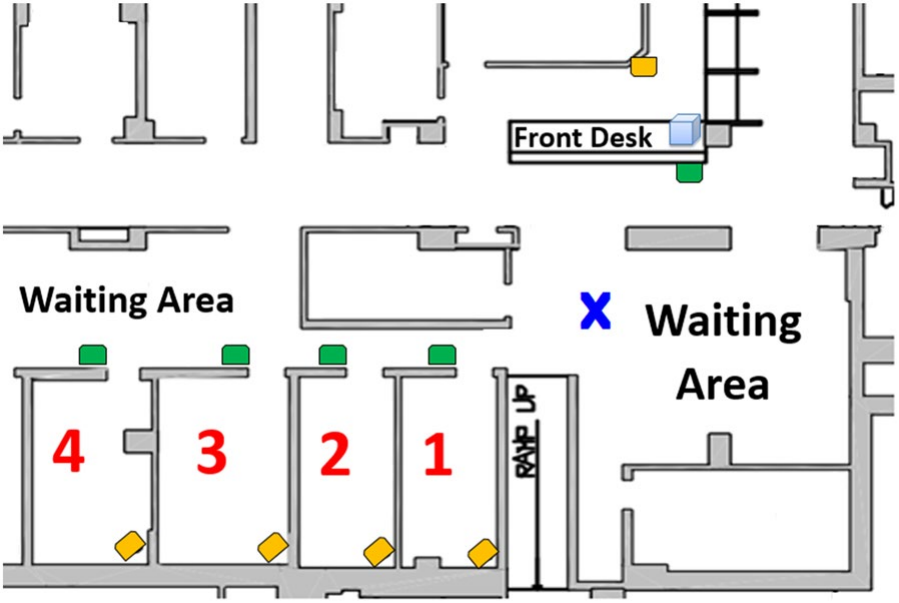
Schematic of General Eye Service Clinic: Patients were tracked with RFID and IR technologies as they moved through the clinic. The *orange shapes* designate the locations of the IR receivers and the *green shapes* designate the locations of the RFID readers. The *blue box* designates the location of the return bin. The *blue* “X” designates the location from which the investigator manually tracked patients. Four exam rooms were used for this trial, labeled 1 through 4 in *red*. The front desk where check-in and checkout took place is labeled. Waiting areas are labeled

The custom-built RFID readers were installed outside exam rooms and at the front desk, and IR receivers were installed inside the exam rooms, in waiting areas and at the front desk (Fig. 4). Because only a portion of the clinic was used for this study, some participants were tracked through one monitored exam room, some through multiple monitored exam rooms, and some through no monitored exam rooms, depending on the particular participant. All measurements included front desk traffic for check-in and checkout.

**Fig. 5:**
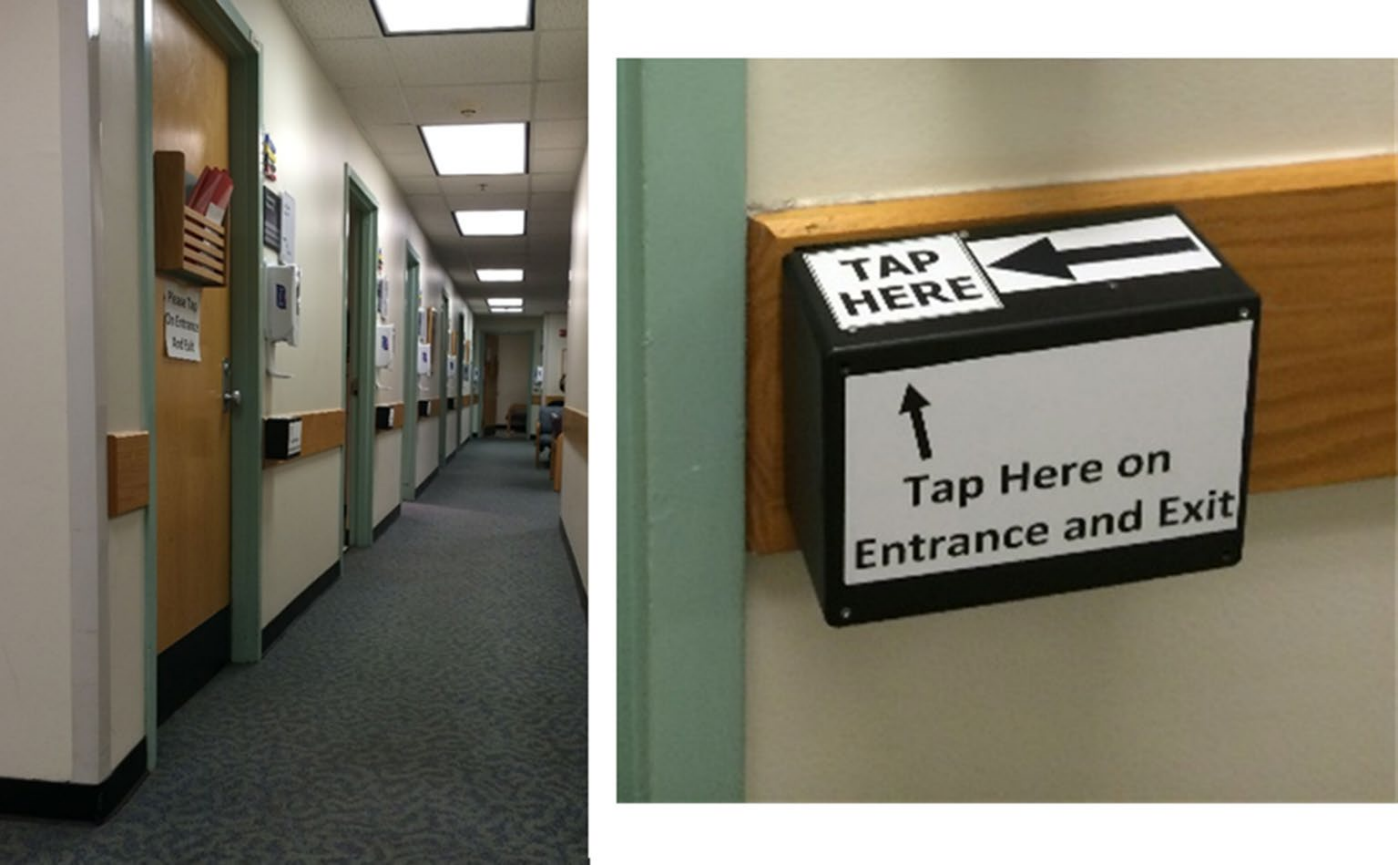
RFID system in the clinic: custom-made RFID receivers were installed outside of clinic exam rooms with reminder signs instructing patients to swipe before and after entrance

One hundred twelve ophthalmology outpatients being seen in the Johns Hopkins Wilmer Eye Institute General Eye Service were enrolled sequentially under Institutional Review Board-approved guidelines. Using a script, an investigator instructed participants to simply wear their IR tag and to swipe the provided RFID proximity card at readers before and after passing through each clinic room. Patients were not told to follow any particular paths so their flow mimicked real-world situations. This instruction was given at the front desk with a check-in swipe demonstration at that location. Although scripting was designed to mirror real-world instruction provided by front desk staff, data collected at check-in for both IR and RFID methods were not used because of the potential confounding effects of the consenting process. Additionally, reminder signs instructing patients to swipe before and after entrance were placed on the doors of the clinic (Fig. 5). No further instruction was given to patients in order to simulate real-world adherence of the technology. At checkout, patients swiped one final time at the front desk reader and dropped off their tags in a labeled return bin.

While in the clinic, participants were simultaneously tracked by RFID and IR technologies while an investigator (SV) manually recorded the times at which patients moved in and out of each tracked area. Investigator-recorded times were established as the standard against which the other methods were compared.

The IR system necessitated installation of a custom wireless network with RF access points ($1395 per access point, 5 total), one central network access point ($1002), open area receivers ($530 per receiver, 4 total), exam room receivers ($370 per receiver, 2 per room, 23 total), a designated wireless router ($65), a designated computer ($1200), and a software license ($9500). Additionally, each IR tag cost $38. With installation covering 25 rooms in the clinic, the total cost of the IR system per tracked location was $1440.88 (Table 1).

**Table 1:** IR tracking system expense list

Item	Price
RF access points (x5)	$1395 each
Central network access point	$1002
Open area receivers (x4)	$530 each
Exam room receivers (x23)	$370 each
Designated wireless router	$65
Designated computer	$1200
Software license	$9500
Total	$36022
Cost per room/area	*$1440.88*
IR tag cost	*$38*

IR itemized expense sheet: the total cost of the commercial IR system came to $1440.88 per each of the 25 rooms/zones with installation. Although only five of the rooms were used for this study, the real world costs per room are approximated using all rooms with installation in order to divide fix costs appropriately

The RFID system involved custom-built readers installed outside exam rooms. The system was made of eleven parts: a Raspberry Pi Computer ($30), an RFID sensor ($49.50), a project enclosure box ($9), a USB wireless adapter ($8.65) an SD card ($5), a power adapter ($3.46), an externally powered USB hub ($10), Industrial fastening strips ($2), an LED light with resistors and jumper wire ($1), eight screws ($1) and a double-output 11,200 mAh USB battery pack with cables ($23). The total cost of parts for each reader came to $142.61 and each proximity card cost $1 (Table 2). One reader per exam room and one reader for front desk check-in/check-out was needed.

**Table 2:** RFID tracking system expense list

Item	Price
Raspberry Pi Computer	$30
RFID sensor	$49.50
Project enclosure box	$9
USB wireless adapter	$8.65
SD card	$5
Power adapter	$3.46
Externally powered USB hub	$10
Industrial fastening tape	$2
LED with resistors and jumper wire	$1
Double-output 11200mAh USB battery	$23
Screws (x8)	$1
Cost per room/area	*$142.61*
IR tag cost	*$1*

RFID itemized expense sheet: the total cost of the custom-built RFID receiver was $142.61. This was also the cost of setup per tracked location because only one box was needed per area. Note that these costs do not involve labor or licensing expenses because they are not applicable

## Results

As implemented, the percentage of successfully acquired timestamps was significantly higher with the RFID system (83.7 %) than the IR system (75.4 %, p < 0.01). An important cause of the error for the IR system was found to be a signal interruption by the front desk to IR signals between participant-worn tags and the front desk receiver. The front desk IR receiver only captured 50.9 % of events compared with 85.4 % capture with RFID (p < 0.01, OR = 5.75 compared to the IR system). Excluding the front desk data (110 events), the rate of successfully acquired timestamps by the IR system improved to 94.4 % compared to 82.4 % with the RFID method (p = 0.002; OR = 3.83 compared to the RFID system).

The IR system also experienced some false positives. This occurred six times with five different tags and three different receivers. During the false positive events, tags in the return bin were somehow detected in exam rooms a significant distance away. The cause of these false positives was not determined. These signals represented a 2.3 % false positive rate for the IR system (n = 6).

Both RFID and IR methods recorded accurate times when compared to manual recordings by an investigator. The mean difference between RFID and stopwatch patient tracking times was 12.9 s (P = 0.47). The mean difference between IR and stopwatch patient tracking times was 20.9 s (p = 0.13). Differences of this magnitude are expected given the manual method serving as the “gold standard” and are not significant when compared to the amount of time spent in clinic overall.

## Discussion

In our evaluation of patient tracking systems, a low-cost, custom-built system utilizing passive RFID technology performed better than a commercial system utilizing IR. Because the IR system lost about half of the participants’ check-out events, it was unable to provide information on how long patient stays ultimately turned out to be. Only after the evaluation was conducted could we determine that the IR system required re-engineering to properly acquire signals at the front desk. With those front desk timestamps excluded, both RFID and IR methods were effective at providing patient flow information.

The efficacy of RFID for use in clinic settings had not been certain. As a tracking method that involves swiping of a proximity card, RFID relies on user participation, which may reduce the rate of correctly acquired timestamps. Patients may forget to swipe cards, may be unable to understand instruction or may find swiping too inconvenient for participation. Nonetheless, with the reminder signs and instruction used in this study, the RFID methodology obtained an adherence of 83.7 %.

Conversely, the commercial IR technology is designed to be passive for the patient, thereby decreasing the risk of patient non-adherence with tracking. Investigators noticed that most loss of event capture tended to be due to either the tag flipping backwards and losing signal or patients not wearing the tag as instructed. This manifested as IR signals that cut in and out frequently, but because only changes in IR tag-receiver interactions are needed to determine localization, this was ultimately found to have a very minor impact on the final data output given the percent of events successfully captured by the system with setup corrections.

In addition to these unavoidable but minor contributors to signal loss, significant but preventable data loss was also found with the IR technology. At the end of the study, it was found that about half of the signals at checkout were dropped because the standing-level front desk height interrupted IR signals between the tag and the front desk receiver. An adjustment to the front desk receiver was required to correct the signal loss.

Similar system failures have been reported before with IR [5, 15–19]. For example, one study that used IR in the emergency room setting found that close proximity of receivers in triage areas and waiting rooms resulted in ambiguous localization of patients in those locations [19]. This risk of RTLS failure may not be unique to IR technology. A review of 23 hospitals using a variety of RTLS technologies found that 35 % had systems with poor accuracy and only 26 % were given high ratings of accuracy or functionality, with no particular technology outperforming others [17]. Therefore, this study’s findings as well as those of previous literature emphasize the importance of methodical assessment of RTLS technologies after installation, particularly among systems with many parts and signals whose setup is unique in each setting.

Despite its system error, the IR technology does allow for more applications than the RFID technology. Though patient tracking was analyzed in this study, the IR infrastructure that was set up could also be used for asset tracking, proximity alarming and notifications. However, capitalizing on such large amounts of data may require expert analysis and infrastructure more readily available at larger institutions.

Additionally, the IR methodology also costs notably more than the RFID method. At a cost of $1440.80 per recording area (Table 1), the IR method may be more appropriate for larger projects and long-term use. Larger projects would also dissipate the IR technology’s significant fixed costs like software licensing and a custom wireless network setup. As implemented, the RFID technology did not incur similar costs (Table 2); however, it did require time and effort to build. Investigators with limited knowledge in technology development may find such tasks challenging. Furthermore, it is important to note that while adding considerable expense, the licensing costs of a commercial IR system include manufacturer technical support and services.

In contrast to IR technologies, the RFID method was more affordable and portable at the cost of being slightly less reliable when system failures are corrected (83.7 % adherence for RFID vs 94.4 % adherence for IR after system failure correction). The RFID receivers were wireless and battery operated, so they were reusable in other settings and could be moved easily on demand without significant costs. The data the RFID system collected were also only timestamps, card ID and sensor ID, allowing for simpler though more limited data collection. Therefore, RFID methods may be more appropriate for smaller projects involving more focused questions or lower budget situations.

To make patient tracking technology more accessible to smaller practices, affordability must be a consideration. As shown in other studies implementing this technology [19], tags are occasionally lost because patients can leave the clinic forgetting that they had them on, potentially adding unanticipated ongoing costs [22]. Identifying methods to reduce tag loss or reduce tag costs will be important considerations. In this study, however, very careful monitoring of patients by investigators resulted in only one lost tag. Nevertheless, more cost-benefit analyses would be beneficial to determine the efficacy of the system in various clinic settings.

Although this study provides the largest non-industry-funded results to validate RTLS in real-world healthcare settings using human measurements as a reference, it is still limited in scope and scale. Our results span only 6 days of recordings and 252 events. Upon completion of the study, investigators were unable to conduct interventions to the IR tracking system to evaluate whether corrections to the system could overcome its front desk signal failures because operations management had contracts for the system to be moved and installed elsewhere at the hospital immediately after the study.

Furthermore, the results of this study may not be generalizable to all clinic settings. Although the General Eye Service is typical for a busy, large ambulatory clinic, not all clinics are similar. Participants were coming for general ophthalmology care and therefore had particular profiles and illness levels. Studies in more healthcare settings are needed to determine how these technologies fare in different environments.

Despite these shortcomings, these findings nevertheless reveal that IR and RFID technologies are comparable in utility. Although RFID has an increased risk of non-adherence compared to IR when set up correctly, both systems may be similarly useful for obtaining pictures of patient flow and guiding cost-saving interventions.

## Conclusions

With proper care and attention toward setup, both RFID and IR methods are equally effective at providing patient flow information. While IR methods can be more reliable, more affordable RFID options may still be useful for data acquisition, particularly among clinics with more limited budgets.

## Abbreviations

RFID: radio frequency identification
IR: infrared
RTLS: real time locating system
RF: radiofrequency

## References

[1] Perez MM, Cabrero-Canosa M, Hermida J, Garcia LC, Gomez DL, Gonzales GV, Herranz IM (2012). Application of RFID technology in patient tracking and medication traceability in emergency care. J Med Syst.

[2] Larson G. Shortest average wait times for doctors in major cities increased one minute year over year. Vitals. 2014.

[3] Medical Practice Wait Times and Patient Satisfaction. Press Ganey. 2010. http://www.pressganey.com/Documents_secure/Medical%20Practices/White%20Papers/Keep_Me_Waiting.pdf. Accessed 5 Dec 2014.

[4] Doran GT, Miller AF, Cunningham JA (1981). There’s a S.M.A.R.T. way to write management’s goals and objectives. Manag Rev (AMA Forum).

[5] Boulos MNK, Berry G (2012). Real-time locating systems (RTLS) in healthcare: a condensed primer. Int J Health Geogr.

[6] Karunaratne LA. Real-time locating systems in agriculture: technical possibilities and limitations. Thesis. Uppsala University; 2010.

[7] Krohn R (2008). The optimal RTLS solution for hospitals. Breaking through a complex environment. J Healthc Inf Manag.

[8] Ng TM (2015). From “Where I Am” to “Here I Am”: accuracy study on location-based services with IBeacon Technology. HKIE Trans.

[9] Gao T, Greenspan D, Welsh M, Juang RR, Alm A. Vital signs monitoring and patient tracking over a wireless network. In: 2005 IEEE Engineering in Medicine and Biology 27th Annual Conference. 2005. 10.1109/IEMBS.2005.1616352 17282121

[10] Wang B, Toobaei M, Danskin R, Ngarmnil T, Pham L, Pham H. Evaluation of RFID and Wi-Fi technologies for RTLS applications in Healthcare Centers. In: Proceedings of PICMET. 2013. p. 2690–703.

[11] Cobbley B. Easing patient flow. Easing patient flow for RTLS. Health Facilities Management, 01 Dec. 2011. Web. 2015. 22329123

[12] The Endless Potential of RTLS—Health Management Technology. Health Management Technology. Wasp Barcode Technologies, 24 June 2013. Web. 2015.

[13] Poshywak J. Fighting infection with RTLS—health management technology. Health Management Technology. General Manager, TeleTracking Technologies, 26 Jan. 2015. Web. 2015. 26211228

[14] Kotzen M. N.J. health system saves $1.2 Million—health management technology. Health Management Technology, 24 July 2013. Web. 2015. 24015488

[15] Marjamaa RA, Torkki PM, Torkki MI, Kirvel OA (2006). Time accuracy of a radio frequency identification patient tracking system for recording operating room timestamps. Anesth Analg.

[16] Okoniewska B, Graham A, Gavrilova M, Wah D, Gilgen J, Coke J, Burden J, Nayyar S, Kaunda J, Yergens D, Baylis B, Ghali WA (2012). Multidimensional evaluation of a radio frequency identification Wi-fi location tracking system in an acute-care hospital setting. J Am Med Inform Assoc.

[17] Fisher JA, Monahan T (2012). Evaluation of real-time location systems in their hospital contexts. Int J Med Informatics.

[18] Curran K, Furey E, Lunney T, Santos J, Woods D, Mccaughey A (2011). An evaluation of indoor location determination technologies. J Location Based Serv.

[19] Miller MJ, Ferrin DM, Flynn T, Ashby M, White KP, Jr., Mauer MG. Using RFID technologies to capture simulation data in a hospital emergency department. In: Proceedings of Winter Simulation Conference, Monterey, California. p. 1365–371.

[20] Sun PR, Wang BH, Wu F (2008). A new method to guard inpatient medication safety by the implementation of RFID. J Med Syst.

[21] Christe B, Rogers R, Cooney E (2010). Analysis of the impact of a radiofrequency identification asset-tracking system in the healthcare setting. J Clin Eng.

[22] Cao Q, Jones DR, Sheng H (2014). Contained nomadic information environments: technology, organization, and environment influences on adoption of hospital RFID patient tracking. Inf Manag.

